# Racial Differences in Psychosocial Outcomes Among Prostate Cancer Survivors: Insights From the All of Us Research Program

**DOI:** 10.7759/cureus.110267

**Published:** 2026-06-04

**Authors:** Johnny Wang, Hannah Cho, Angelina T Wang, Narmina Khanmammadova, Karim Hanna, David I Lee

**Affiliations:** 1 Department of Urology, University of California, Irvine, Irvine, USA; 2 School of Medicine, University of California, Irvine, Irvine, USA

**Keywords:** cancer survivorship, depression, prostate cancer (pca), psychosocial outcomes, racial disparity, socioeconomic factors

## Abstract

Introduction

Prostate cancer (PCa) survivorship is associated with substantial psychological distress, yet racial differences in mental health outcomes are incompletely understood. We used multivariable logistic regression models to evaluate associations between race and post-diagnosis depressive episodes and patient-reported psychosocial outcomes, including quality of life, mental health, and social satisfaction, among PCa survivors in a diverse national cohort.

Methods

We conducted a cross-sectional study using the National Institutes of Health (NIH) All of Us Research Program Controlled Tier Dataset (version 8). Adults with electronic health records (EHR)-documented PCa who completed surveys after diagnosis were included. Non-Hispanic White (NHW) and Black participants were compared. Primary outcomes included depressive episodes after diagnosis and patient-reported quality of life, mental health, and social satisfaction. Multivariable regression models adjusted for age, time since diagnosis, socioeconomic factors, medical comorbidities, and baseline depression.

Results

Among 4,524 PCa survivors, 3,965 (87.6%) were NHW and 559 (12.4%) were Black. Black participants were younger (median 66.8 years vs 72.6, p<0.001) and experienced greater socioeconomic disadvantage (219 (39.2%) with <$25,000 annual household income vs. 262 (6.6%), p<0.001). Depressive episodes were more common among Black individuals (97 (17.4%) vs 509 (12.8%)). On univariate analysis, Black race was associated with higher odds of depressive episode (OR 1.31, 95% CI 1.07-1.59, p=0.008), but this association did not persist after adjustment (adjusted OR 0.80, 95% CI 0.60-1.06, p=0.119). In contrast, NHW participants reported worse psychosocial outcomes, with higher proportions reporting fair or poor quality of life, mental health, and social satisfaction. Black race was associated with lower odds of worse self-reported primary outcomes on univariate analysis; after adjustment, this association persisted for quality of life (adjusted OR 0.75, 95% CI 0.61-0.92, p=0.005).

Conclusion

Racial differences in EHR-documented depression were largely explained by socioeconomic factors, while patient-reported psychosocial outcomes were similar or more favorable among Black individuals. Divergence between clinical diagnoses and patient-reported measures highlights challenges in accurately assessing psychological distress across populations.

## Introduction

Prostate cancer (PCa) is the most common cancer among men in the United States (US) [1]. Though early detection and more optimized treatment have decreased mortality, many patients experience substantial psychological distress after diagnosis. Data suggest that roughly one in six men with PCa report significant depressive symptoms and nearly one in ten report recent suicidal ideation [2].

Existing evidence suggests that Black patients experience worse cancer-related outcomes than their White counterparts in this population. Black men are more likely to present with advanced disease and have greater cancer-specific mortality [3,4]. Though Black men may be more vulnerable to adverse psychosocial outcomes, it remains a challenge to disentangle the complex social and biological factors that contribute to mental health. Varying trends have been described in this population, including increased risk of depression and increased risk of mortality associated with depression but also increased resilience [5,6]. Prior epidemiologic studies have reported higher crude rates of depression among Black patients compared with White patients, although this association may attenuate or reverse after adjustment. More data to guide understanding of racial disparities is necessary to inform survivorship care.

The role of race in shaping psychosocial wellbeing among PCa survivors is incompletely characterized. Studies across other malignancies including breast, colorectal, and head and neck cancer have revealed significant racial disparities, highlighting the need to again evaluate whether a consistent trend can be established for PCa [7-9]. We investigated racial differences in diagnosed depressive episodes and self-reported measures of quality of life, mental health, and social satisfaction among individuals with PCa in a diverse, population-based cohort using multivariable regression models adjusted for demographic and socioeconomic factors.

## Materials and methods

Study design

We conducted a cross-sectional analysis using the National Institutes of Health (NIH) All of Us Research Program Controlled Tier Dataset version 8 [10], a national data repository that includes participant-reported outcome surveys, standardized electronic health record (EHR) information, wearable device data, and genomic data. This dataset is designed to promote representation of traditionally underrepresented groups in biomedical research. Participant-level data were obtained and analyzed in compliance with the All of Us Data Use and Registration agreement. This study adhered to the Strengthening the Reporting of Observational Studies in Epidemiology (STROBE) guidelines [11].

Study cohort

Participants included adults over 18 years of age distributed across all 50 states. As of early 2026, more than 874,000 participants were enrolled nationwide, of whom 52% identify as White and 46% are over the age of 60.

**Figure 1:**
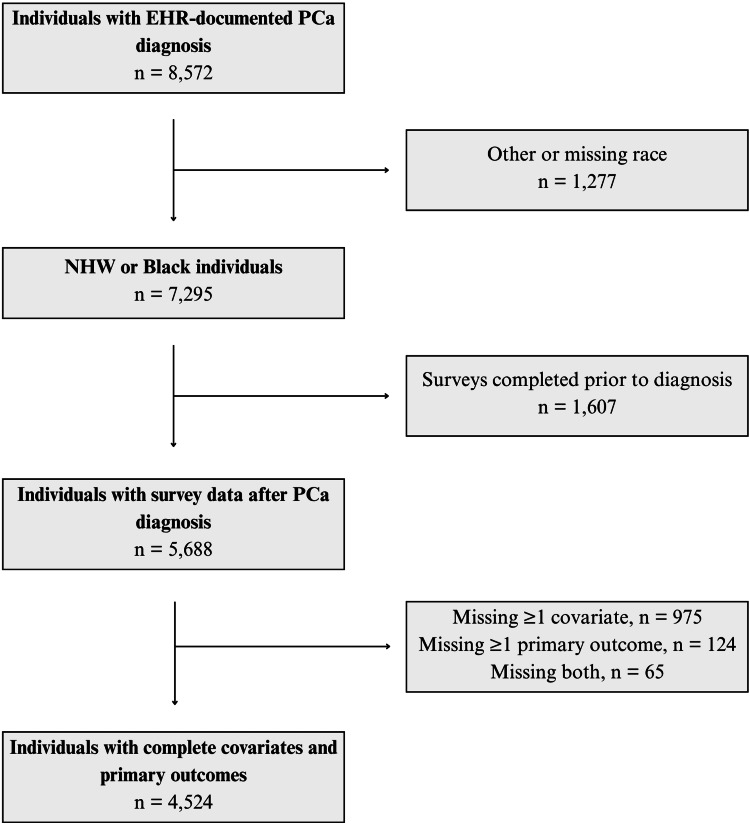
Flow diagram of study cohort criteria EHR: electronic health record; PCa: prostate cancer; NHW: non-Hispanic White

The All of Us Researcher Workbench was used for cohort selection. A flow diagram is included in Figure 1. PCa patients were identified using an EHR-documented diagnosis of primary malignant neoplasm of the prostate (SNOMED CT (Systematized Nomenclature of Medicine - Clinical Terms) code 93974005). Eligible individuals were required to have completed surveys pertaining to demographics (“The Basics”), socioeconomic factors (“Social Determinants” and “Healthcare Access & Utilization”), and mental and physical health (“Overall Health”) after PCa diagnosis. Non-Hispanic White (NHW) or Black race was determined using the “Demographics” concept set. Those with other or missing race were excluded. Clinical variables such as hypertension, diabetes, obesity, and preexisting mood disorders were extracted from EHR data. Individuals with missing key covariates or survey responses required for primary outcomes were excluded.

Outcomes and definitions

The primary outcomes included EHR-documented depressive episode after PCa diagnosis (International Classification of Diseases (ICD) 10 code F32 [12]), as well as patient-reported outcomes of quality of life, mental health, and social satisfaction. Responses to the latter survey-based outcomes were natively coded as “poor”, “fair”, ”good”, ”very good”, and “excellent” but grouped as “poor/fair”, “good”, and “excellent/very good” for analysis. In our multivariable analysis, survey-based outcomes were modeled as ordinal variables, with higher categories representing worse self-reported outcomes. Secondary outcomes included characteristics and perceptions of social support, discrimination, stress/resilience, healthcare experiences, and substance use.

Statistical analysis

Participant characteristics were summarized using proportions for categorical variables and medians with interquartile ranges (IQRs) for continuous variables. Comparisons were drawn using chi-square or Wilcoxon rank-sum tests as appropriate. Univariate and multivariate binary and ordinal logistic regression models were used to evaluate associations between race and primary outcomes. Multivariate models were adjusted for age, time since diagnosis, socioeconomic indicators, medical comorbidities, and baseline depression. Covariates were selected a priori based on prior literature, conceptual relevance to social determinants of health and psychosocial outcomes, and data availability within the All of Us dataset. Sensitivity analyses demonstrated that regression results were unchanged with alternative coding of the survey outcomes. Due to data missingness, secondary outcomes were analyzed descriptively only. All statistical tests were two-sided with 0.05 as the threshold for significance. Analyses were performed in RStudio version 4.2.3 (R Foundation for Statistical Computing, Vienna, Austria, https://www.R-project.org/) within the All of Us Researcher Workbench cloud environment.

## Results

Cohort characteristics

A total of 4,524 individuals with prostate cancer met inclusion criteria, including 3,965 (87.6%) NHW and 559 (12.4%) Black individuals (Table 1). Black participants were younger at the time of survey completion (median 66.8 vs 72.6 years, p<0.001) and had a shorter time since diagnosis (median 4.08 vs 4.93 years, p<0.001). In terms of sociodemographic characteristics, there were significant differences in education level, marital status, employment, and income. Notably, 3,637 (91.8%) NHW individuals held undergraduate or more advanced degrees, compared to 380 (68%) Black individuals. The proportion of Black individuals living without a partner was more than twice that of NHW individuals (199 (35.6%) vs. 675 (17.0%)). There was also major economic disparity, with 219 (39.2%) Black individuals reporting a household income of <$25,000 per year compared to only 262 (6.6%) among NHW individuals. In terms of medical comorbidities, Black patients were more likely to have type 2 diabetes mellitus (164 (29.3%) vs. 763 (19.2%), p<0.001) and hypertension (361 (64.6%) vs. 2,038 (51.4%), p<0.001) at baseline, but there were no differences in baseline major depression or anxiety disorders.

**Table 1:** Demographics and covariates Cells with counts less than 20 were suppressed according to the All of Us Data and Statistics Dissemination Policy [13]. GED: General Educational Development

Variable		Non-Hispanic White	Black	p-value	χ² (df)	Effect Size (Cramér’s V)
Age (years), mean ± SD		72.6 ± 7.9	67.1 ± 8.7	<0.001	-	-
Time since diagnosis (years), mean ± SD		6.4 ± 5.5	5.5 ± 5.1	<0.001	-	-
Highest education level, n (%)	Advanced degree	1707 (43.1%)	97 (17.4%)	<0.001	347.99 (3)	0.277
	College	1930 (48.7%)	283 (50.6%)			
	High school/GED	321 (8.1%)	165 (29.5%)			
	Less than high school	<20	<20			
Marital status, n (%)	Married/living with partner	3290 (83.0%)	360 (64.4%)	<0.001	107.27 (1)	0.155
	Divorced/separated/widowed	675 (17.0%)	199 (35.6%)			
Employment status, n (%)	Employed for wages or self-employed	1125 (28.4%)	130 (23.3%)	<0.001	346.98 (3)	0.277
	Out of work	80 (2.0%)	33 (5.9%)			
	Unable to work	85 (2.1%)	100 (17.9%)			
	Retired	2675 (67.5%)	296 (53.0%)			
Household income ($), n (%)	<25,000	262 (6.6%)	219 (39.2%)	<0.001	563.71 (3)	0.353
	25,000-50,000	1798 (45.3%)	196 (35.1%)			
	50,001-100,000	1270 (32.0%)	119 (21.3%)			
	>100,000	635 (16.0%)	25 (4.5%)			
Comorbidities, n (%)	Type 2 diabetes mellitus	763 (19.2%)	164 (29.3%)	<0.001	30.03 (1)	0.082
	Hypertension	2038 (51.4%)	361 (64.6%)	<0.001	33.64 (1)	0.087
Baseline diagnosis of major depressive disorder, n (%)		166 (4.2%)	32 (5.7%)	0.12	2.41 (1)	0.025
Baseline diagnosis of generalized anxiety disorder, n (%)		86 (2.2%)	<20	0.54	0.38 (1)	0.011

**Figure 2:**
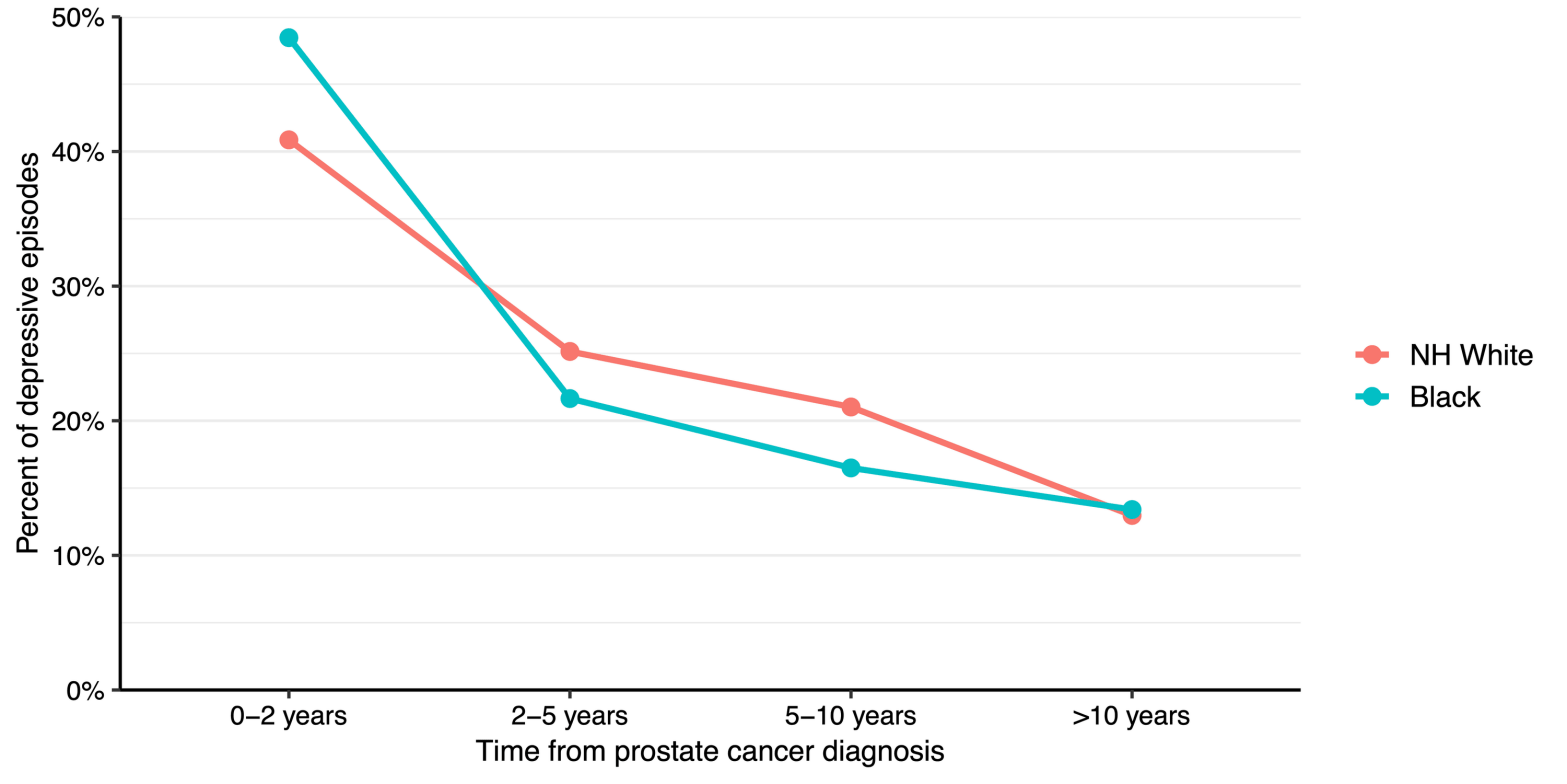
Temporal distribution of depressive episodes by time from cancer diagnosis NH: non-Hispanic

On Spearman correlation analysis, older age was weakly positively correlated with worse scores across all three primary survey outcomes (Table 2). Time since diagnosis also demonstrated a weak correlation with worse quality of life (ρ=0.040, p=0.008) but not with the remaining measures. Figure 2 shows the distribution of depressive episodes across different time points after PCa diagnosis. A large proportion of depressive episodes occurred within two years after cancer diagnosis for both groups (48.5% and 40.9% for Black and White individuals, respectively). There was no difference in the overall temporal distribution across race groups (χ2 = 2.355, p=0.502).

**Table 2:** Spearman correlation analysis

Exposure	Outcome	Correlation coefficient (ρ)	p-value
Age	Quality of Life	0.126	<0.001
	Mental Health	0.12	<0.001
	Social Satisfaction	0.117	<0.001
Time since diagnosis	Quality of Life	0.04	0.008
	Mental Health	0.016	0.28
	Social Satisfaction	0.022	0.14

Primary outcomes

Primary outcomes included EHR-documented depression, quality of life, mental health, and social satisfaction (Table 3). Depressive episodes were more common among Black individuals (97 (17.4%) vs. 509 (12.8%), p<0.001). On univariate analysis, Black race was associated with higher odds of a depressive episode (OR 1.43, 95% CI 1.12-1.81, p=0.003). However, this association was no longer statistically significant after multivariable adjustment (adjusted OR (aOR) 0.8, 95% CI 0.6-1.06, p=0.119).

**Table 3:** Comparison of primary outcomes based on race among PCa survivors PCa: prostate cancer; NHW: non-Hispanic White

Outcome		NHW	Black	Unadjusted OR (95% CI)	p-value	Adjusted OR (95% CI)	p-value	χ² (df)	Effect Size (Cramér’s V)
EHR-documented depressive episode		509 (12.8%)	97 (17.4%)	1.31 (1.07-1.59)	0.0081	0.8 (0.6-1.06)	0.119	8.61 (1)	0.044
In general, would you say your quality of life is:	Fair/poor	2892 (72.9%)	247 (44.2%)	0.28 (0.24-0.32)	<0.001	0.75 (0.61-0.92)	0.005	213.47 (2)	0.217
	Good	822 (20.7%)	206 (36.9%)						
	Excellent/very good	251 (6.3%)	106 (19.0%)						
In general, how would you rate your mental health, including your mood and your ability to think?	Fair/poor	2952 (74.5%)	327 (58.5%)	0.41 (0.36-0.48)	<0.001	1.01 (0.82-1.25)	0.927	86.29 (2)	0.138
	Good	760 (19.2%)	143 (25.6%)						
	Excellent/very good	253 (6.4%)	89 (15.9%)						
In general, how would you rate your satisfaction with your social activities and relationships?	Fair/poor	2687 (67.8%)	269 (48.1%)	0.41 (0.36-0.47)	<0.001	0.99 (0.81-1.21)	0.903	90.88 (2)	0.142
	Good	866 (21.8%)	176 (31.5%)						
	Excellent/very good	412 (10.4%)	114 (20.4%)						

In contrast, survey-based outcomes demonstrated worse self-reported psychosocial wellbeing among NHW individuals. Most NHW individuals reported fair/poor quality of life, mental health, and social satisfaction (2,892 (72.9%), 2,952 (74.5%), 2,687 (67.8%), respectively), compared with 247 (44.2%), 327 (58.5%), and 269 (48.1%) among Black individuals. Correspondingly, Black race was associated with lower odds of worse quality of life (OR 0.29, 95% CI 0.25-0.35, p<0.001), mental health (OR 0.46, 95% CI 0.38-0.55, p<0.001), and social satisfaction (OR 0.44, 95% CI 0.37-0.53, p<0.001). After adjustment for covariates, Black race remained associated with lower odds of worse reported quality of life (aOR 0.76, 95% CI 0.62-0.93, p=0.008).

Secondary outcomes

Tables 4, 5 summarize secondary outcomes in the domains of social support, discrimination, stress/resilience, healthcare utilization, and substance use. General trends indicated decreased social support. Specifically, Black individuals were less likely to have someone to help them if they were confined to bed (119 (63.0%) most or all of the time vs. 1,840 (74.6%)), to take them to the doctor if needed (133 (70.7%) most or all of the time vs. 1,969 (80.1%)), to have a good time with (114 (64.0%) most or all of the time vs. 1,729 (74.2%)), and who loves them and makes them feel wanted (122 (67.0%) most or all of the time vs. 1,897 (78.6%)). For perceived discrimination, Black individuals were less likely to report being treated with less courtesy and less respect than others in general (79 (42.5%) never or less than once a year vs. 401 (16.2%); 78 (42.4%) vs. 355 (14.5%)) but more likely to do so in healthcare settings; some subgroup results were not resported due to cell counts below All of Us dissemination threshold. There were no differences in degree of stress or resilience, as well as no difference in reported visits with mental health professionals. Alcohol use was more common among NHW individuals (1,046 (27.2%) four or more drinks per week vs. 56 (11.2%)), whereas cigarette smoking was far more common among Black individuals (77 (26.3%) every day vs. 93 (5.0%)).

**Table 4:** Comparison of patient-reported outcomes of social support, discrimination, and stress/resilience by race Cells with counts less than 20 were suppressed according to the All of Us Data and Statistics Dissemination Policy [13].

Parameter	Outcome		Non-Hispanic White	Black	p-value	χ² (df)	Effect Size (Cramér’s V)
Social support	How often do you feel isolated from others?	Rarely/never	1940 (80.7%)	148 (82.7%)	0.803	0.47 (2)	0.013
		Sometimes	67 (2.8%)	<20			
		Often	396 (16.5%)	<50			
	How often do you have someone to help you if you were confined to bed?	None or a little of the time	337 (13.7%)	32 (16.9%)	0.0007	14.48 (2)	0.074
		Some of the time	290 (11.8%)	38 (20.1%)			
		Most or all of the time	1840 (74.6%)	119 (63.0%)			
	How often do you have someone to have a good time with?	None or a little of the time	249 (10.7%)	28 (15.7%)	0.0118	8.88 (2)	0.059
		Some of the time	353 (15.1%)	36 (20.2%)			
		Most or all of the time	1729 (74.2%)	114 (64.0%)			
	How often do you have someone who understands your problems?	None or a little of the time	354 (14.6%)	29 (15.7%)	0.281	2.54 (2)	0.031
		Some of the time	399 (16.4%)	38 (20.5%)			
		Most or all of the time	1674 (69.0%)	118 (63.8%)			
	How often do you have someone who loves you and makes you feel wanted?	None or a little of the time	295 (12.2%)	32 (17.6%)	0.0012	13.38 (2)	0.072
		Some of the time	223 (9.2%)	28 (15.4%)			
		Most or all of the time	1897 (78.6%)	122 (67.0%)			
Discrimination	In your day-to-day life, how often are you treated with less courtesy than other people are?	Less than once a year/never	401 (16.2%)	79 (42.5%)	<0.001	88.52 (2)	0.183
		A few times a year/a few times a month	2039 (82.6%)	101 (54.3%)			
		At least once a week	29 (1.2%)	<20			
	In your day-to-day life, how often are you treated with less respect than other people are?	Less than once a year/never	355 (14.5%)	78 (42.4%)	<0.001	104 (2)	0.199
		A few times a year/a few times a month	2061 (84.2%)	100 (54.3%)			
		At least once a week	32 (1.3%)	<20			
Stress/resilience	In the last month, how often have you felt nervous and “stressed”?	Almost never/never	1475 (67.3%)	114 (67.9%)	0.954	0.02 (2)	0.003
		Sometimes	716 (32.7%)	54 (32.1%)			
		Often	<20	<20			
	In the last month, how often have you felt difficulties were piling up so high that you could not overcome them?	Almost never/never	1883 (88.5%)	141 (88.7%)	1	0.001 (2)	0.001
		Sometimes	244 (11.5%)	<20			
		Often	<20	<20			

**Table 5:** Comparison of patient-reported outcomes in healthcare access and substance use by race Cells with counts less than 20 were suppressed according to the All of Us Data and Statistics Dissemination Policy [13].

Parameters	Outcome		NHW	Black	p-value	χ² (df)	Effect Size (Cramér’s V)
Healthcare access	Have you visited a mental health professional (psychiatrist, psychologist, psychiatric nurse, or clinical social worker) within the last 12 months?		386 (15.7%)	37 (18.0%)	0.434	0.779 (1)	0.017
	How often did your health care providers tell or give you information about your health and health care that was easy to understand?	None or a little of the time	10 (0.4%)	<20	0.174	4.6 (2)	0.039
		Some of the time	83 (3.0%)	<20			
		Most or all of the time	2706 (96.7%)	222 (94.1%)			
	How often did your health care providers ask for your opinions or beliefs about your medical care or treatment? For example, what kind of tests, procedures, or medications you prefer.	None or a little of the time	262 (9.5%)	20 (8.8%)	0.124	4.356 (2)	0.038
		Some of the time	708 (25.6%)	45 (19.7%)			
		Most or all of the time	1797 (64.9%)	163 (71.5%)			
	How often are you treated with less courtesy than other people when you go to a doctor's office or other health care provider?	Never/rarely	2344 (95.0%)	155 (81.6%)	<0.001	90.41 (2)	0.184
		Sometimes	68 (2.8%)	<50			
		Most of the time/always	54 (2.2%)	<20			
	How often are you treated with less respect than other people when you go to a doctor's office or other health care provider?	Never/rarely	2318 (95.0%)	156 (82.9%)	<0.001	71.2 (2)	0.165
		Sometimes	74 (3.0%)	<50			
		Most of the time/always	48 (2.0%)	<20			
Substance use	Do you now smoke cigarettes every day, some days, or not at all?	Not at all	1715 (92.0%)	171 (58.4%)	<0.001	260.94 (2)	0.348
		Some days	56 (3.0%)	45 (15.4%)			
		Every day	93 (5.0%)	77 (26.3%)			
	How often did you have a drink containing alcohol in the past year?	Never	682 (17.8%)	142 (28.4%)	<0.001	106.07 (4)	0.156
		Monthly or less	758 (19.7%)	145 (29.0%)			
		2 to 4 drinks per month	678 (17.6%)	105 (21.0%)			
		2 to 3 drinks per week	678 (17.6%)	52 (10.4%)			
		4 or more drinks per week	1046 (27.2%)	56 (11.2%)			

## Discussion

In this population-based cohort of PCa survivors, racial differences in EHR-documented depressive episodes were largely attenuated after adjustment for socioeconomic factors. In contrast, patient-reported outcomes demonstrated better self-reported quality of life, mental health, and social satisfaction among Black participants, with the association persisting for quality of life after adjustment. These findings suggest that socioeconomic and structural factors may play an important role in shaping observed racial differences in mental health outcomes. However, given the cross-sectional design and potential residual confounding, causal interpretation regarding the role of race itself should be made cautiously and considered within broader social contexts.

Men with PCa are at risk for greater psychological distress compared to the general population. Overall, a 2021 meta-analysis by Brunckhorst et al. estimated the pooled prevalence for depressive disorders at 5.8%, with clinically significant depressive symptoms identified in 17.1% [2]. In an analysis of the Surveillance, Epidemiology, and End Results (SEER) Utah cancer registry, Hu et al. found an increased risk of depressive disorder among all PCa survivors, with the greatest risk at 0-2 years after diagnosis [14]. Though the cross-sectional nature of this study precludes statements of incidence, we also observed a majority of depressive episodes occurring in the immediate post-diagnosis period, suggesting the importance of this time in identifying those at highest risk. Separately, a large national cohort study in Sweden demonstrated diverging patterns based on cancer risk group, with a more than two-fold elevated risk of major depression and death by suicide in the high-risk PCa population persisting more than 10 years after diagnosis, but the same associations in the low- or intermediate-risk PCa groups were only observed in the first year after diagnosis [15].

Given treatment-related side effects and social stigma, a PCa diagnosis can have profound implications for mental health [16,17]. Conversely, poor mental health is an underrecognized comorbidity that may adversely impact cancer outcomes. Depression itself is associated with an up to 74% increase in PCa-specific mortality [18]. In addition, PCa patients experiencing greater psychological distress also have higher rates of healthcare utilization, including outpatient clinic visits and emergency room visits [19,20]. Addressing mental health needs has system-level impacts on comprehensive and cost-effective PCa care.

Prior studies evaluating mental health outcomes among prostate cancer survivors have produced heterogeneous findings even within similarly defined study cohorts. This variability is illustrated by two large population-based studies in the US. In a 2021 study of men with localized prostate cancer within the Veterans Health Administration, African American men were 15% more likely than White men to be diagnosed with depression [6]. By comparison, a 2025 analysis of SEER-Medicare data found that non-Hispanic Black individuals were 24% less likely than NHW individuals to develop depression 5-10 years after diagnosis [21]. In the current study, there was no significant association between Black race and incident depressive episodes in this population after adjustment, suggesting that observed differences in mental health outcomes may be influenced more by social determinants than by race itself. This is also consistent with growing recognition that clinical outcomes are shaped by the interplay of biological, clinical, and social factors, highlighting the importance of accounting for population heterogeneity when interpreting outcome differences.

These findings should also be interpreted in the context of the “Black-White depression paradox”, a well-described epidemiologic observation in which Black Americans have similar or lower rates of depression despite greater exposure to stressors [22-24]. Numerous explanations have been proposed, though the underlying causes are likely multifactorial. Artefactual explanations include inconsistent operationalization of depression, differential performance of diagnostic instruments, and somatization of depressive symptoms [25-27]. The divergence between chart and survey outcomes in the current study corroborates the idea that differences in measurement and classification are indeed impactful on observed patterns. 

Alternatively, etiologic explanations include internal processes, such as increased resilience or reliance on coping behaviors like substance use, yet these ideas contrast with the current findings [28-30]. Resilience is commonly linked to stronger social support, but this sample suggests that Black individuals have equal or better psychosocial outcomes in spite of worse social support [31,32]. Furthermore, we show that alcohol use, particularly heavier use, was actually more common among White individuals. In terms of external factors, lower mental illness screening rates and healthcare access in Black communities are likely to contribute [33,34]. Data show that Black patients have different, and often worse, healthcare experiences, which is supported by the observation that Black individuals in this sample were significantly more likely to report maltreatment in the healthcare setting. Together, these findings suggest that observed racial differences in mental health outcomes may reflect differences in healthcare engagement and structural factors beyond intrinsic differences in psychological vulnerability, which again supports the notion that timely and effective intervention is needed in this area.

Limitations

Several limitations warrant consideration. First, cross-sectional design precludes causal inference within this study. Second, oncologic variables such as disease stage, treatment, and treatment-related adverse effects were not consistently available within the dataset. Although certain oncologic variables may be queried from EHR data, these are subject to incomplete capture, inconsistent coding, and misclassification. Therefore, we were unable to reliably assess the influence of disease severity, treatment type, or treatment-related side effects on psychosocial outcomes. Given that these factors may influence psychological distress and survivorship outcomes, their absence may have introduced residual confounding when evaluating associations between race and psychosocial outcomes. While survey completion dates are available, exact diagnosis dates, treatment start dates, and treatment completion dates cannot be reliably queried from the EHR. As a result, we were unable to confidently determine whether surveys were completed immediately after diagnosis, during treatment, or after treatment. Additionally, while survey measurements add granularity to the above findings, reliance on surveys also introduces selection and voluntary response bias. Finally, though survey questions in the All of Us dataset are derived from previously validated instruments, individual survey questions reflect global self-assessment rather than validated scores, limiting direct comparability to other epidemiologic studies. As such, these measures may not capture the full dimensionality of mental health, quality of life, or social satisfaction and should be interpreted as broad self-reported health domains rather than clinically validated diagnoses or scale-based symptom burden. Reliance on EHR diagnosis codes to identify depression diagnoses may also introduce misclassification. Despite these limitations, the study leverages a demographically diverse national cohort and integrates both EHR-derived diagnoses and patient-reported measures, allowing complementary assessment of mental health outcomes across multiple domains.

## Conclusions

In this national cohort of PCa survivors, racial differences in EHR-documented depression were largely attenuated after adjustment for socioeconomic factors, while patient-reported psychosocial outcomes were similar or more favorable among Black individuals. The observed divergence between chart-documented diagnoses and patient-reported measures further highlights challenges in accurately identifying psychological distress across populations. Improving access to mental health screening in real-world practice and developing more robust tools for clinical and epidemiologic investigation will be important to ensure that psychosocial needs are addressed equitably among men with PCa.
